# Personal

**Published:** 1911-10

**Authors:** 


### PERSONAL

(The editors would be glad to receive items for this department from any physician, whether a subscriber or not. In the future, we shall rely largely upon notices of removal, change of office hours and personal notes of travel, and the like, furnished by the physician himself.)

Dr. Irving M. Snow has returned from Europe.

Dr. J. A. Ragone spent September in the Muskoka region.

Dr. C. A. Tyler of Alden returned early in September from Toronto.

Dr. Ray H. Johnson returned early in September from a month's outing.

Dr. Rudolf C. Miller visited friends in and near Toronto, early in September.

Dr. John R. Gray spent the latter part of August and the first of September in Ontario.

Dr. W. H. Marcy returned about the middle of September from his summer home at Webster, Mass.

Dr. A. VanderVeer of Albany, has been spending the summer at Camp Alkmaar, Big Moose Lake, Herkimer County, N. Y.

Dr. F. Park Lewis, who has spent the summer in Europe, arrived in Buffalo a few days ago, having sailed on La Lorraine.

Dr. Daniel A. Eiseline of Shortsville, a graduate of the University of Buffalo, has been nominated for Coroner by the Republicans of Ontario County.

Dr. James L. Gallagher has been reëlected Supreme Medical Examiner for the Atlantic, Central and Southern States, by the convention of the Ancient Order of Foresters.

DR. M. M. SMITH, of Dallas, Editor of the *Texas Medical News*, paid us a friendly call in September. He is a bright, brainy, practical man and, while he was too courteous to offer advice, we managed to get from him many useful hints which will benefit our readers.

---

DR. GEORGE R. CRITCHLOW returned from his vacation early in September.

